# PRED^TAP^: a system for prediction of peptide binding to the human transporter associated with antigen processing

**DOI:** 10.1186/1745-7580-2-3

**Published:** 2006-05-23

**Authors:** Guang Lan Zhang, Nikolai Petrovsky, Chee Keong Kwoh, J Thomas August, Vladimir Brusic

**Affiliations:** 1 Institute for Infocomm Research, 21 Heng Mui Keng Terrace, 119613, Singapore; 2 School of Computer Engineering, Nanyang Technological University, 6397984, Singapore; 3 Department of Diabetes and Endocrinology, Flinders Medical Centre/Flinders University, Flinders Drive, Bedford Park, Adelaide, 5042, Australia; 4 Division of Biomedical Sciences, Johns Hopkins Medicine in Singapore and Department of Pharmacology and Molecular Sciences, Johns Hopkins School of Medicine, Baltimore, MD 21205, USA; 5 School of Land and Food Sciences and the Institute for Molecular Bioscience, University of Queensland, Brisbane QLD 4072, Australia

## Abstract

**Background:**

The transporter associated with antigen processing (TAP) is a critical component of the major histocompatibility complex (MHC) class I antigen processing and presentation pathway. TAP transports antigenic peptides into the endoplasmic reticulum where it loads them into the binding groove of MHC class I molecules. Because peptides must first be transported by TAP in order to be presented on MHC class I, TAP binding preferences should impact significantly on T-cell epitope selection.

**Description:**

PRED^TAP ^is a computational system that predicts peptide binding to human TAP. It uses artificial neural networks and hidden Markov models as predictive engines. Extensive testing was performed to valid the prediction models. The results showed that PRED^TAP ^was both sensitive and specific and had good predictive ability (area under the receiver operating characteristic curve Aroc>0.85).

**Conclusion:**

PRED^TAP ^can be integrated with prediction systems for MHC class I binding peptides for improved performance of *in silico *prediction of T-cell epitopes. PRED^TAP ^is available for public use at [1].

## Background

Peptides that bind major histocompatibility complex (MHC) class I molecules serve as recognition targets for cytotoxic CD8^+ ^T cells (CTLs). The major function of CTLs is recognition and destruction of infected (e.g. viruses, bacteria, parasites or fungi), mutated (e.g. cancer), or foreign (e.g. transplants) cells. CTLs recognize short antigenic peptides (T-cell epitopes) presented by MHC class I molecules that mainly originate from degradation of cytosolic proteins. Intracellular antigen processing pathways determine the selectivity of peptides which are available for binding to MHC class I molecules and are thereby important targets of CTL responses [2].

MHC class I antigen processing pathway steps include proteosomal cleavage of proteins into shorter peptides, translocation of peptides into the endoplasmic reticulum (ER) by TAP, optional ER trimming by aminopeptidases, insertion of peptides into the binding groove of MHC molecules, and transport of peptide/MHC complexes to the cell surface for presentation to CTLs [3]. TAP is a transmembrane protein responsible for the transport of antigenic peptides into the ER. TAP demonstrates peptide binding selectivity and the affinity of a particular peptide for TAP influences the probability of its presentation by MHC class I molecules. Peptides that are 8–16 amino acids long and have sufficient binding affinity are efficiently translocated by TAP into the ER, while longer peptides may be transported but with lower efficiency [4]. Human TAP (hTAP) is a heterodimer that has two subunits hTAP1 and hTAP2. TAP belongs to the ATP-binding cassette transporters and each subunit protein has one transmembrane domain and one ATP-binding binding domain. The genes for human TAP1 and TAP2 are located in the MHC II locus of chromosome 6 and comprise 10 kb each [5]. A more detailed description of function, structure, expression of TAP can be found in [6].

The efficiency of TAP-mediated translocation of a peptide is proportional to its TAP-binding affinity [7,8]. Mutations, such as premature stop codons, or deletions of either hTAP1 or hTAP2 impair peptide transport into ER and result in a significant reduction of surface expression of peptide/MHC complexes [9]. TAP deficient cells have low cell-surface HLA class I expression shown to range from 10% (HLA-A2) to 3%, (HLA-B27 and -A3) [10]. The majority of the peptides presented by HLA class I on cell surface are thus dependent on TAP.

Identification of T-cell epitopes is a highly combinatorial problem. The diversity of human immune responses to T-cell epitopes originates from two sources – high allelic variation of the host (both HLA molecules and T-cell receptors) and high variation of target antigens, particularly those derived from viruses. Computational models are routinely used for pre-screening of potential T-cell epitopes and minimization of the number of necessary experiments. Most developments have focused on modeling and prediction of peptide binding to MHC molecules [see [11]]. Amongst computational models of peptide binding to hTAP that have been developed are binding motifs [7], quantitative matrices [12-14], artificial neural networks (ANN) [12,15], and support vector machines (SVM) [16]. Combined computational methods that integrate multiple critical steps – proteasome cleavage, TAP transport, and MHC class I binding have been proposed as a supporting methodology for prediction of high probability targets for therapeutic peptides and vaccines [17]. Several combined computational applications of models of antigen processing and presentation have been reported [18-22]. Testing results indicate that these predictions produce a lower incidence of false positives and reduce the number of experiments required for identification of T-cell epitopes. However, these combined predictions need to be taken with a dose of caution. Alternative pathways for both proteolytic degradation [23] and TAP transport [24] have been reported. In some cases TAP-deficient individuals have normal immune responses [25], suggesting that TAP-independent immune responses are sufficient to provide effective protection from some intracellular pathogens. Nevertheless, the proteasome-TAP-MHC class I pathway is responsible for 90–97% of expression of peptide/MHC Class I complexes and therefore is critical for the identification of target epitopes for immunotherapies and vaccines.

We developed PRED^TAP^, a computational system that predicts peptides binding to hTAP. It uses ANN and hidden Markov models (HMM) as predictive engines. Extensive testing was performed to validate the prediction models and ensure that PRED^TAP ^is both sensitive and specific. PRED^TAP ^is available for public use at [1].

## Materials and methods

### Training dataset

There are 493 nonamer peptides in the training dataset (Table 1) [12,15]. A single duplicate peptide was removed from the data set reported in the original references. The binding scores range from zero to ten. Scores 7–10 denote high peptide/TAP binding affinity, 5–6 moderate binding affinity, 3–4 low binding affinity and scores 0–2 denote non-binding. The dataset is available in the supplementary materials.

**Table 1:** Number of peptides in the training dataset

Binding Affinity	Number of peptides
0	26
1	52
2	48
3	48
4	53
5	55
6	40
7	87
8	61
9	16
10	7

Sum	493

### Artificial Neural Network

3-layer backpropagation ANN models (in-house software) were used for the development of the PRED^TAP ^server. The learning method was error backpropagation with a sigmoid activation function. The inputs to the ANN were the binary strings representing nonamer peptides. There are twenty naturally-occurring amino acids encoded by the standard genetic code. Each amino acid in a nonamer peptide can be encoded as a binary string of length 20 with a unique position set to "1" and other positions set to "0", resulting in a binary string of length 180 to represent the nonamer. For example the first two amino acids, by alphabetic order, alanine (A) and cysteine (C) are encoded by 10000000000000000000 and 01000000000000000000 respectively, and the last amino acid tyrosine (Y) is encoded by 00000000000000000001. The outputs were binding scores ranging from zero to ten. The higher the score, the higher the possibility of the peptide being a TAP binder. Two ANN architectures were used, 180-2-1 and 180-1-1. The maximum number of the ANN training cycles was set to 300. The training was repeated for four times, and four sets of weights were obtained. The value of momentum was 0.5 and of learning rate 0.2. The error threshold for stopping training was 0.01.

### Hidden Markov Model

HMMs have been applied successfully in prediction of HLA class I-binding peptides [26,27]. An HMM is defined by a finite set of states representing possible states of the modeled system. Some of these states may be directly observable, but some are not, and are denoted as hidden. Biological problems are often sequential and HMM frequently utilize sequential ordering of system states. A change (transition) of the system from one state to another is governed by statistical regularities. The probability distribution of the system states can be estimated from the data. In the present study, we used a first-order HMM, in which the current system state is determined only by the preceding state, as described in [26].

### Cross-validation

Cross-validation is a method for error rate estimation. It implements a simple idea: the dataset of size n samples is partitioned into two parts, the model parameters are estimated using one set and the goodness-of-fit criterion evaluated on the second set. The cross-validation estimates the goodness-of-fit criterion. Cross-validation tends to overfit when selecting a correct model – it may choos an overly-complex model for the given dataset. There is some evidence that for model selection multifold cross-validation, where more than one samples are deleted form the training set in each comparison, performs better than a simple leave-one-out cross-validation[28]. In our experiments, 10-fold cross-validation was performed to evaluate the performance of the classifiers.

### Prediction performance measurement

The predictive performance of the models was evaluated by sensitivity (SE) and specificity (SP) measures. Sensitivity, SE = TP/(TP+FN), indicates percentage of correctly predicted binders, where TP stands for number of true positive predictions (experimental binder predicted as binder) and FN stands for number of false negative predictions (experimental binder predicted as non-binder). Specificity, SP = TN/(TN+FP), indicates percentage of correctly predicted non-binders, where TN stands for number of true negative predictions (experimental non-binder predicted as binder) and FP stands for number of false positive predictions (experimental non-binder predicted as binder). For the studied problem, we consider values of SP >0.8 useful in practice.

The receiver operating characteristic (ROC) curve analysis provided a measure for overall prediction accuracies of prediction models [29]. The ROC curve is generated by plotting SE against (1-SP) for various classification thresholds. As a rough guide, the area under ROC (Aroc) value 1.0 represents a perfect prediction, values 0.9 to 1.0 represent excellent accuracy, 0.8 to 0.9 represent good accuracy, 0.7 to 0.8 represent marginal accuracy, 0.5 to 0.7 represents poor accuracy, while 0.5 represent predictions that indicate random choice [29].

The prediction performance of PRED^TAP^(ANN & HMM) was compared with that of publicly available predictive systems, TAPPred (SVM & cascade SVM) [16] and SVMTAP [19]. Three proteins, human papillomavirus type 16 E6 (P03126) with experimentally identified HLA-A3 binders [30], E7 (P03129) with a single HLA-A3 binder [30] peptides and human cancer antigen KM-HN-1 (NP_689988.1) with three HLA-A24 restricted T-cell epitopes [31], were used and the predicted TAP binders were compared with the HLA binding peptides.

### Normalization of prediction scores

Brusic *et al*. [15] showed that ANN models were skewed with a tendency to center-shift prediction of both very low and very high TAP binders. To obtain prediction scores evenly distributed in the range 0–10, we have implemented prediction score normalization. The raw prediction scores produced by HMM methods are not within the range 0–10. Score mapping is also necessary to bring final prediction scores within the range 0–10. The mapping of scores was done according to equation:

scoren = (score - scoremin) / (scoremax - scoremin)   × 10

*score*_*n *_denotes the normalized score, *score *denotes the raw prediction score, *score*_*min *_and *score*_*max *_denote the minimum and maximum values of the raw scores. The values for *score*_*min *_and *score*_*max *_were obtained using extensive simulation. More than 5000 randomly selected nonamer peptides were used for prediction using the ANN/HMM models. Since the testing data contains large number of nonamer peptides, the highest and lowest predicted score from the testing data were taken as reasonable maximum and minimum scores for normalization.

### Implementation

The web interface of PRED^TAP ^uses a set of Graphical User Interface forms. The interface was built using a combination of Perl, CGI and C programs. PRED^TAP ^has been implemented in the SunOS 5.9 UNIX environment.

## Model validation

Assessment of predictive accuracy was carried out for three subsets of peptide binders: 1) all binders including low, moderate and high binders were considered as positive samples, and all non-binders as negative samples (referred to as the LMH set); 2) moderate and high binders were considered as positive samples, all non-binder and low binders as negative samples (referred to as the MH set), and 3) only high binders were considered as positive samples, with all other peptides as negative samples (referred to as the H set). The Aroc values of ANN and HMM models are shown in Table 2. All models showed very good predictive performance. For MH set and H set, ANN models showed excellent performance with Aroc values above 0.9. For LMH set, the Aroc values of ANN models are above 0.85. ANN with structure 180-2-1 showed slightly better performance than that of ANN with structure 180-1-1. Thus ANN with structure 180-2-1 was adopted in our system. The performance of HMM model is also good with Aroc values above 0.85.

**Table 2:** Performance assessment of ANN/HMM models using 10-fold cross-validation

ANN 180-2-1	H	MH	LMH
1st run	0.95	0.95	0.89
2nd run	0.95	0.94	0.88
3rd run	0.95	0.94	0.88

ANN 180-1-1	H	MH	LMH

1st run	0.93	0.94	0.87
2nd run	0.92	0.92	0.86
3rd run	0.93	0.94	0.88

HMM	H	M	L

1st run	0.9	0.9	0.87
2nd run	0.89	0.9	0.87
3rd run	0.89	0.9	0.86

The specificity *vs*. sensitivity plot of the ANN prediction model for prediction can be viewed at supplementary materials A [1]. The specificity/sensitivity plot of the HMM prediction model can be viewed at supplementary materials B [1].

**Figure 1:**
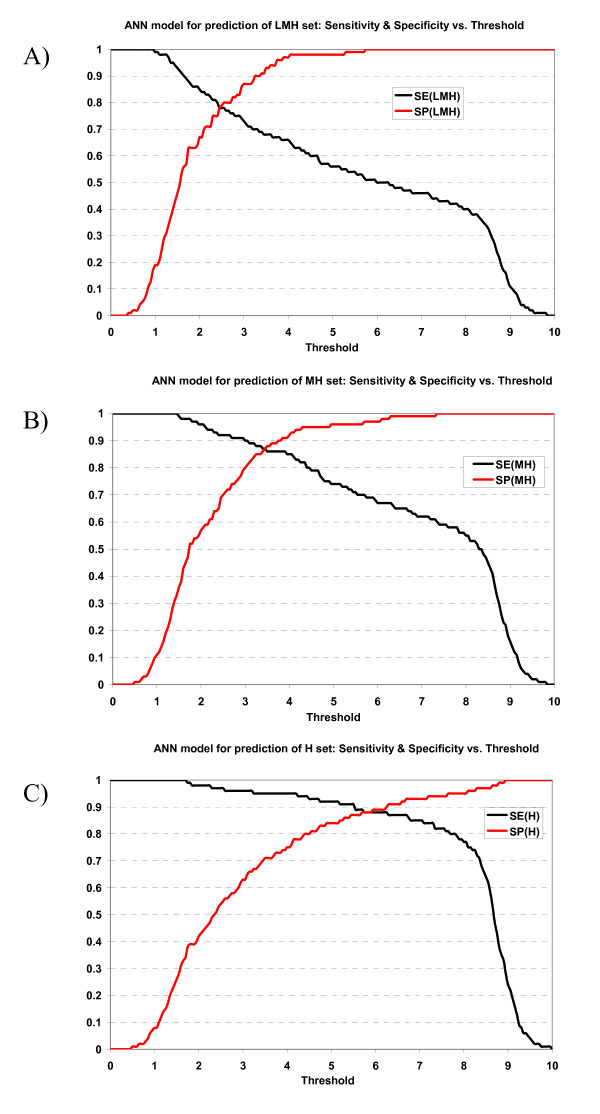
Plot of sensitivity and specificity of ANN model against thresholds in 10-fold cross-validation. The ANN model for prediction of A) LMH set, B) MH set, and C) H set.

**Figure 2:**
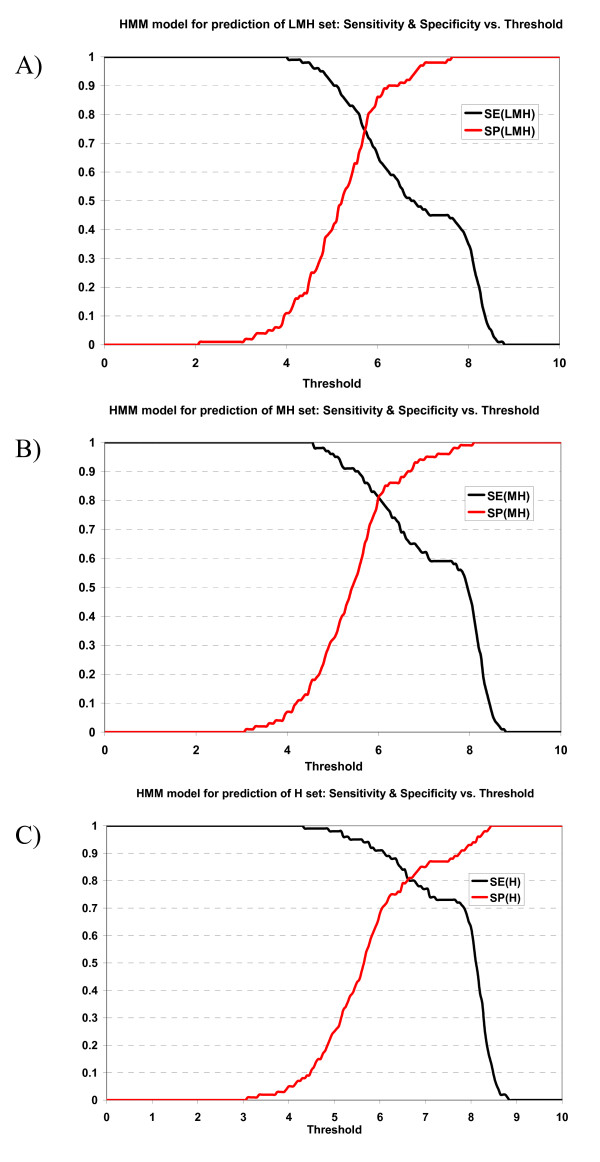
Plot of sensitivity and specificity of HMM model against thresholds in 10-fold cross-validation. The HMM model for prediction of A) LMH set, B) MH set, and C) H set.

Sensitivities and specificities of ANN and HMM models at various thresholds (based on normalized scores) in 10-fold cross-validation experiments are shown in Figures 1 and 2. We selected the normalized score of 6.0 as a reasonable selection threshold, with peptides with scores ≥ 6.0 predicted as TAP binders. In Table 3, the sensitivities and specificities of ANN and HMM models at the selection threshold 6.0 are shown. ANN model managed to correctly predict 88% of high binders at the cost of 11% of false positives (the 11% also includes moderate and low-affinity binders); 67% moderate and high binders with 3% false positives in the MH set, and 50% of all binders (low, moderate and high) with practically no false positives (Table 3A). The specificities of ANN model for all three sets (LMH, MH and H sets) are high (1.00, 0.97, 0.89 respectively), which indicates that 6.0 is a stringent selection threshold and the false positive rate is very low at this threshold. At threshold 6.0, HMM model managed to correctly predict 91% of high binders with 32% false positives, 81% moderate and high binders with 19% false positives, and 66% of all binders (low, moderate and high) with 14% false positives (Table 3B). The specificity of the HMM model for LMH set was 0.86, higher than that of MH set which was 0.81. The specificity of the HMM model for MH set is much higher than that of H set, which was 0.68. It implies that HMM model was able to select binders (low, moderate and high binders) with low false positive rate, but it failed to categorize them into subgroups – low, moderate or high binders.

**Table 3:** Sensitivities and specificities of ANN and HMM models at the selection threshold 6.0

Threshold	ANN	SE	SP
6.0	LMH	0.50	1.00
	MH	0.67	0.97
	H	0.88	0.89

Threshold	HMM	SE	SP

6.0	LMH	0.66	.86
	MH	0.81	0.81
	H	0.91	0.68

To evaluate the predictive power of the methods, the dataset was partitioned into a training set containing two thirds of the data points randomly selected and a testing set containing the remaining one third of data points. The tests were conducted three times for each ANN and HMM methods. The Aroc values of ANN and HMM models are shown in Table 4. Despite smaller training datasets being used ANN models continued to show excellent performance with Aroc values above 0.9 for H and MH sets and good performance with Aroc values above 0.85 for LMH set. The performance of HMM model is also good with Aroc values above 0.85. The performance of HMM dropped slightly with Aroc values above 0.85 for H and MH sets and above 0.80 for LMH set.

**Table 4:** Performance assessment of ANN/HMM models when the dataset was partitioned into two parts with the training dataset containing two thirds of the data points randomly selected and the testing set containing the remaining one third of data points

ANN 180-2-1	H	MH	LMH
1st run	0.91	0.92	0.85
2nd run	0.96	0.95	0.90
3rd run	0.94	0.91	0.87

HMM	H	M	L

1st run	0.88	0.88	0.86
2nd run	0.86	0.88	0.83
3rd run	0.91	0.9	0.82

## Comparison to other predictive systems

Since PRED^TAP^, TAPPred and SVMTAP were built using the same set of   training data [12,15], independent data sets must be used to test and   compare their prediction performance. Rather, we compared the predictions on human papillomavirus type 16 E6 and E7 and the amino acid positions of top 5% predicted TAP binders were shown in Tables 5 and 6. Half of the experimental HLA-A3 binders overlapped predicted TAP-binders. As suggested by previous studies [15,32] HLA-A3 binding peptides have high affinity to TAP, in agreement with our results. The SVMTAP, TAPPred (SVM), and PRED^TAP ^(ANN & HMM) predicted similar sets of TAP-binding peptides while TAPPred (cascade SVM) predictions were different (Table 5). A single HLA-A3 binder from E7 protein did not overlap any of predicted TAP binders except for TAPPred (cascade SVM) (Table 6). Again, the TAPPred (cascade SVM) predicted completely different set of peptides as compared to the other four predictors.

**Table 5:** Amino acid position of top 5% predicted TAP binders in Human papillomavirus type 16 E6 (P03126) by SVMTAP, TAPPred and PRED^TAP^. The positions marked by "+" were selected by four prediction models. The positions marked by "*"were selected by three prediction models. The experimentally identified HLA-A*0301 binders are ^1^7–15, ^2^33–41, ^3^42–50, ^4^59–67, ^5^75–83, ^6^89–97, ^7^93–101, and ^8^125–133). The predictions in the table marked by ^1–8 ^are within 16-mers containing respective HLA-A*0301 binders

SVMTAP	TAPPred (SVM)	TAPPred (Cascade SVM)	PRED^TAP ^(ANN)	PRED^TAP ^(HMM)
75^5,+^	53^4,+^	5^1^	75^5,+^	75^5,+^
131^8^	68	60^4^	53^4,+^	46^3,*^
53^4,+^	80^5^	49^3^	46^3,*^	61^4^
150	81^5^	132^8^	68	47^3^
130^8^	75^5,+^	93^7^	83	7^1^
46^3,*^	131^8^	116	59^4^	53^4,+^
134	134	67	42^3^	49^3^
146	51	40^2,3^	130^8^	83^6^

**Table 6:** Amino acid position of the top 5% predicted TAP binders in HPV 16 E7 (P03129) by SVMTAP, TAPPred and PRED^TAP^. The positions marked by "+" were selected by four prediction models and those marked by "*"were selected by three prediction models. The experimentally identified HLA-A*0201 binder is 89–97. ^1^Within a 16-mer containing E7 89–97

SVMTAP	TAPPred (SVM)	TAPPred (Cascade SVM)	PRED^TAP ^(ANN)	PRED^TAP ^(HMM)
49^+^	49^+^	58	50*	49^+^
9*	50*	57	9*	44
50*	17	88^1^	49^+^	43
59	9*	82^1^	48	71
7	59	67	76	3

Three naturally processed peptides from tumor antigen KM-HM-1, namely 196–204, 499–508, and 770–778, are naturally processed by HLA-24 [31]. HLA-A24 binding peptides have been reported as TAP efficient [15,32]. KM-HN-1 protein is 833 amino acids long, and we used top 3% of the predictions (Table 7). Peptide 195–203, which has 8 amino acids overlap to the KM-HN-1196-204, was selected by SVMTAP, TAPPred (SVM) and PRED^TAP ^(ANN & HMM), but not by TAPPred (cascade SVM). Peptide 499–508, was selected by the four methods as a potential 16-mer, also as a 12-mer by PRED^TAP ^(ANN), but not by TAPPred (cascade SVM). It was shown that some peptides are efficiently transported by TAP in their optimal size for MHC class I binding, while some peptides are transported as larger peptides that need further trimming in ER for MHC class I binding [33]. It is likely that peptides 196–204, 499–508, and 770–778, are transported to ER in the longer form and then further trimmed for loading to the HLA-A24 molecules.

**Table 7:** Amino acid position of top 3% predicted TAP binders in the tumor antigen KM-HN-1 (NP_689988.1) by SVMTAP, TAPPred and PRED^TAP^. The positions marked by "+" were selected by four prediction models and those marked by "*"were selected by three prediction models. The predicted TAP-binders in proximity of known T-cell epitopes are designated by ^1^(196–204), ^2^(499–508) and ^3^(770–778)

SVMTAP	TAPPred (SVM)	TAPPred (Cascade SVM)	PRED^TAP ^(ANN)		PRED^TAP ^(HMM)	
Position	Position	Position	Position	Score	Position	Score

660^+^	372^+^	674	195^1,+^	8.15	682	7.01
372^+^	195^1,+^	314	654*	8.09	506^2,+^	6.98
426*	426*	639	372^+^	8.06	372^+^	6.87
195^1,+^	794	249	422	7.94	683	6.83
794	330	530	565	7.41	507^+^	6.65
199	317^+^	525	317^+^	6.53	195^1,+^	6.62
654*	660^+^	325	310	5.97	492	6.44
317^+^	331	206	378	5.59	310	6.41
371	652*	12	468	5.54	660^+^	6.41
110	199	479	763^3^	5.48	16	6.37
760	198	537	337	5.36	468	6.33
198	371	112	426*	5.33	317^+^	6.19
789	654*	93	737	5.16	395*	6.16
705	506^2,+^	626	246	5.04	573	6.12
457	789	470	660^+^	4.98	223	6.12
507^+^	457	141	756	4.96	193	6.09
48	730	483	110	4.95	730	6.09
573	304	71	506^2,+^	4.89	313	6.05
376	565	99	201	4.79	647	6.05
652*	395*	668	507^+^	4.76	510	6.01
395*	760	781	365	4.64	652*	6.01
780	318	57	653	4.55	15	6.01
455	764^3^	579	502^2^	4.52	814	6.01
506^2,+^	63	124	492	4.44	676	5.94
324	507^+^	590	456	4.43	782	5.94

## Using PRED^TAP^

**Figure 3:**
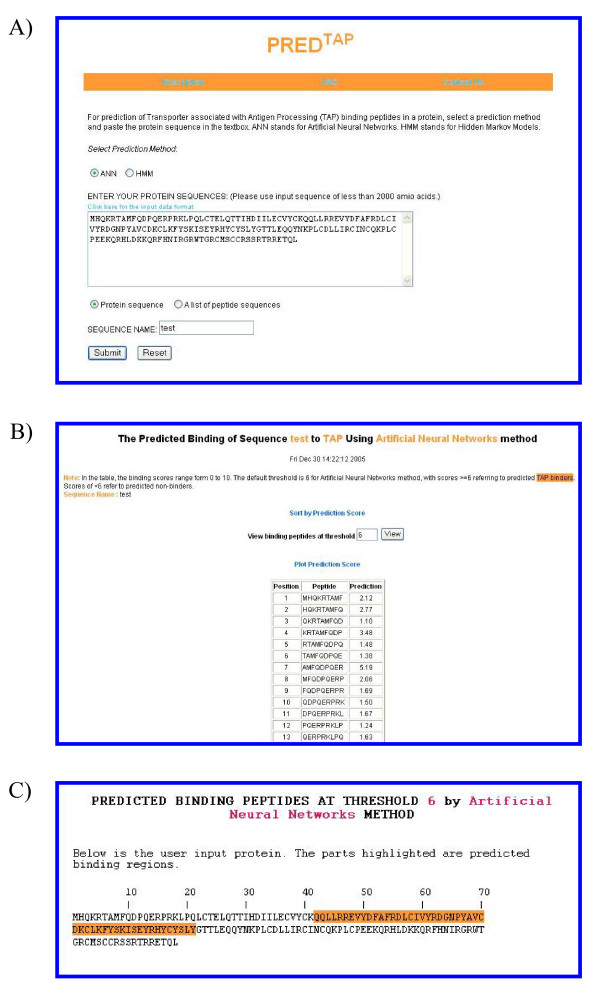
The examples of the output pages of PRED^TAP ^for a single protein. The sequence type chosen is "protein sequence". A) The input page. B) The main result page. The input sequence is decomposed into overlapping 9-mers for prediction of binding scores to TAP. C) Alignment view of the predicted TAP binding regions in the input protein.

To perform predictions using PRED^TAP^, the user needs to paste a protein sequence into the textbox and assign a name to the sequence. The sequence must contain between nine and 2000 amino acids. If the prediction is run with input sequence containing symbols other than 20 amino acid codes (spaces and carriage returns are allowed) or the total sequence length is outside 9–2000 amino acids range, an error message will be displayed and predictions will not be produced. The input can either be a contiguous protein sequence (an amino acid sequence, or FASTA format) or a list of peptides, one per line. The default selection on the webpage is "Protein sequence" (Figure 3A), which means the input sequence is treated as a contiguous protein sequence (carriage returns and line breaks will be ignored). The PRED^TAP ^input processing program decomposes protein sequence (or the list of peptides) into a series of 9-mer peptides overlapping by eight amino acids. Individual 9-mer peptides are then submitted for prediction. Predicted binding scores for all 9-mers are displayed in the result tables (Figure 3B). The 9-mer binding scores are within the range 0–10, the higher the score the higher the probability of peptide being binder. PRED^TAP ^has an option for plotting the binding scores of all the overlapping 9-mer peptides as a graph, in which X axis represents the start position of a 9-mer peptide and Y axis represents the binding score of the 9-mer peptide. The user can sort the peptides by their binding scores and choose to view only predicted binders with binding scores above a certain threshold (Figure 3C).

**Figure 4:**
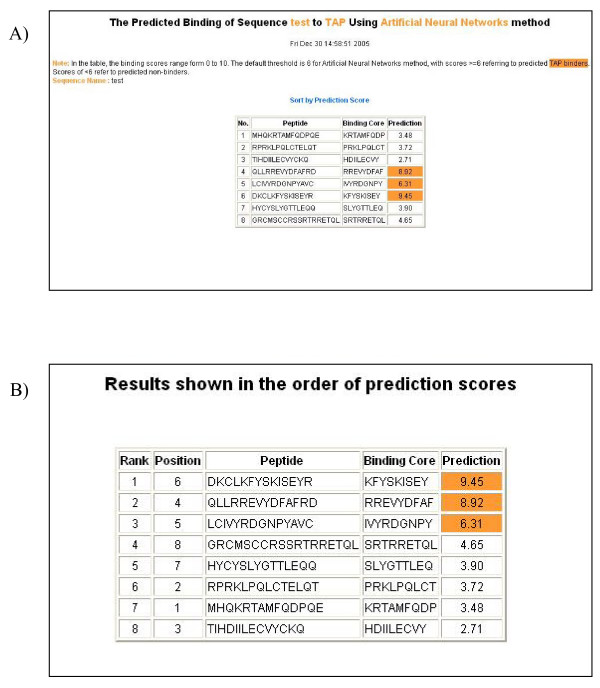
An example of the output pages of PRED^TAP ^for a list of peptides. A) The input page. B) The main result page. All 9-mers in each peptide were submitted for prediction. The predicted binding scores are represented by the highest individual 9-mer binding score of each input peptide. The 9-mer with the highest binding score in each peptide is displayed as "Binding Core" in the result table.

When users select the input sequence type to be "a list of peptide sequences", the input sequences separated by carriage returns or line breaks are treated as different peptides (Figure 4A). All overlapping 9-mers in each peptide are submitted for prediction. In the result tables, predicted binding scores are represented by the highest individual 9-mer binding score within the input peptide. The 9-mer with the highest binding score in each peptide is displayed as "Binding Core" in the result table. The user can sort the peptides by their binding scores (Figure 4B).

## Discussion

We have earlier compared four prediction servers for prediction of H-2K^d ^binding peptides [34]. A 121-amino acid long sequence of the nuclear export protein NS2 from influenza A virus (GenPept accession NP_859033) was searched for 9-mer candidate binders to a mouse MHC molecule H-2K^d ^using four internet-accessible systems. Only three peptides were predicted within the top ten candidates as binders by all four methods. The performance comparison of PRED^TAP ^with SVMTAP and TAPPred (SVM) shows that consensus peptides can be selected by combining predictions. The examples suggested that individual predictions need to be taken with care and predictions may be improved by a consensus of multiple methods. A similar situation may be applicable to TAP predictions. Hence the combination of ANN and HMM predictions in PRED^TAP ^should result in higher specificity (fewer false positives) at the cost of slightly lower sensitivity. The predictions by TAPPred (cascade SVM) appear to be of a limited value.

The combinatorial properties of molecular mechanisms involved in antigen processing and adaptive learning nature of the immune responses limit our ability to fully predict immune responses. Combining experimental and computational techniques improves our ability to decipher complex interactions of the immune system. Computer models are used to complement laboratory experiments and thereby speed up knowledge discovery in immunology. In particular, the number of large-scale laboratory experiments for T-cell epitope mapping can be minimised by the judicious use of experiments aimed at developing and validating computer models. These models can then be used to perform large-scale computer simulations rapidly and inexpensively. The hypotheses generated from these experiments can then be retested in the laboratory to confirm their applicability to real-life immunology. Further work will include both the refinement of computational models and scanning disease-related antigens for peptide sequences that show high probability of processing and presentation. Those peptides that are most likely to be produced by proteasomal cleavage, transported by TAP, and bound by HLA class I molecules are likely to be promising candidates for peptide-based CTL vaccines. The PRED^TAP ^server provides for the prediction of peptide binding by TAP and can be used as a comparison method against other TAP-prediction servers.
